# Lycopene improves autophagy and attenuates carbon tetrachloride-induced hepatic fibrosis in rats

**DOI:** 10.3325/cmj.2023.64.243

**Published:** 2023-08

**Authors:** Wei Li, Yuxin Jiang, Ting-Ting Yu, Wei Hao, Guoguang Wang

**Affiliations:** 1 Department of Pathophysiology, Wannan Medical College, Wuhu, China; 2 Jiaxing University, Jiaxing, China; 3 Experimental Center for Function Subjects, Wannan Medical College, Wuhu, China; Li et al: Lycopene improves autophagy and attenuates carbon tetrachloride-induced hepatic fibrosis in rats

## Abstract

**Aim:**

To evaluate the effect of lycopene on carbon tetrachloride (CCl_4_)-induced hepatic fibrosis and elucidate the underlying mechanism.

**Methods:**

Male rats were randomly assigned to the control group, CCl_4_ group, and lycopene group. The CCl_4_ group was intraperitoneally injected with CCl_4_ twice per week for 12 weeks to induce hepatic fibrosis. The control group was intraperitoneally injected with olive oil. Lycopene was orally administered during CCl_4_ treatment. Body weight and liver weight were recorded. Liver function was assessed. Biomarkers of oxidative stress and inflammatory factors were measured. Histological changes and collagen expression were evaluated. The expression of TGF-β1, α-SMA, HO-1, SIRT 1, REDD1, SHP2, P62, and LC3 in the liver was determined, as well as the levels of phosphorylated NF-κB and IκB α.

**Results:**

Lycopene significantly reduced the liver/body weight ratio, and AST (*P* = 0.001) and ALT levels (*P* = 0.009). It also significantly increased CAT and SOD activities (*P* < 0.001) and decreased MDA content (*P* < 0.001), IL-6 (*P* < 0.001), and TNF-α (*P* = 0.001). Histological analysis demonstrated that lycopene improved lobular architecture and decreased collagen expression. It also decreased the expression of TGF-β1, α-SMA, P62, and SHP2, and increased the ratio of LC3 II/I, as well as Beclin 1 and REDD1 expression. In addition, it reduced NF-κB and IκB-α phosphorylation, and elevated the levels of HO-1, SIRT 1, and PGC 1α.

**Conclusion:**

Lycopene attenuates CCl_4_-induced hepatic fibrosis because of its effect on autophagy by reducing oxidative stress and inflammation.

Due to its high prevalence and high mortality, liver fibrosis is increasingly becoming a threat to public health (1). Liver fibrosis is a result of wound healing after sustained and repeated liver injury, possibly developing into cirrhosis and hepatocellular carcinoma (2,3). The pathogenesis of this condition involves complex mechanisms that are not fully understood. The leading causes of its initiation and progression are oxidative stress and inflammation. In the liver, reactive oxygen species (ROS) generated by exposure to harmful substances (hepatic viruses, medicines, and chemicals) cause acute or chronic liver injury by damaging biomacromolecules such as protein and DNA, and by destroying the hepatic structure (4). Liver fibrosis is associated with the activation of hepatic stellate cells (HSCs) (5). HSCs, insulted by many external factors, can be converted into myofibroblasts (activated HSCs), a process leading to excessive secretion of extracellular matrix (ECM) and driving liver fibrogenesis (6). A key profibrogenic cytokine for the activation of HSCs is transforming growth factor β (TGF-β), which generates α-smooth muscle actin (α-SMA) and ECM, triggering a fibrotic response (7-9). Oxidative stress plays an important role in liver injury caused by multiple factors, such as CCl_4_, obesity, and bisphenol A (10-12). Thus, natural antioxidants are an effective therapeutic strategy for the treatment of liver fibrosis.

Lycopene is a natural bioactive component present in red fruits such as tomatoes, watermelons, and carrots. As a carotenoid, lycopene contains 11 conjugated and two unconjugated double bonds but it cannot be transformed into vitamin A owing to no β-ionone ring structure. Therefore, highly unsaturated lycopene is a potent antioxidant. The ability of lycopene to quench free radicals is 10 times higher than that of α-tocopherol (13). Lycopene protects against oxidative injury to biomacromolecules such as DNA and protein, and alleviates organ damage induced by oxidative stress (14). In our previous study, lycopene ameliorated renal function via inhibiting oxidative stress in diabetic rats (15). Lycopene improves various types of liver injury, such as lipopolysaccharide-induced liver injury (16), titanium dioxide (TiO_2_) nanoparticle-induced damage (17), and liver injury in the liver of aflatoxin B_1_-exposed broilers (18). In addition, it inhibits HSC activation and reduces bisphenol A-induced hepatotoxicity (12,19). However, the effect of lycopene on CCl_4_-induced liver fibrosis has not been investigated. Therefore, in this study, we investigated the effect of lycopene on liver fibrosis.

Autophagy is an endogenous defense mechanism involved in the regulation of cellular homeostasis, cell survival, and apoptosis (20). Various diseases, such as liver diseases, lung injury, and infection, are closely associated with autophagy (20-22). Impairment of autophagy worsens oxidative stress and accelerates liver fibrosis (23), while the activation of autophagy pathway by sirtuin (Sirt) 1 ameliorates liver fibrosis (24). Sirt1, a member of conserved NAD^+^-dependent histone III deacetylases, plays a role in various physiological functions including cell survival, mediation of energy, and tissue regeneration (25-27). Sirt1 also fosters various pathological changes by regulating the inflammatory response and oxidative stress (28,29). Furthermore, Sirt1 expression is elevated by several natural compounds (activators of Sirt 1) (25).

The aim of this study was to investigate the effect of lycopene on CCl_4_-induced hepatic fibrosis, body weight, liver function, and histological changes and explore its possible mechanism of action.

## METHODS

### Material

Lycopene (502-65-8, 98% purity) was purchased from Nanjing Xinkailong Bioengineering Co., Ltd (Nanjing, China). Carbon tetrachloride (CCl_4_) was purchased from Suzhou Baiyu Chemical Co., Ltd (Suzhou, China). Antibodies β-actin, heme oxygenase 1 (HO-1), IκB α, p-IκB α, NF-κB, and p-NF-κB were obtained from Bio Basic Inc. (Markham, ON, Canada). Antibodies SIRT 1, peroxisome proliferator-activated receptor gamma coactivator 1α (PGC-1α), microtubule-associated protein 1 light chain 3B (LC3), P62, Beclin 1, regulated in development and DNA damage responses 1 (REDD1), Src homology 2 domain-containing phosphatase 2 (SHP2), transforming growth factor beta (TGF-β), and α-SMA were purchased from Abcam (Cambridge, MA, USA).

### Experimental animals

Male Sprague-Dawley rats (240-270 g body weight, 8-10 weeks old) were obtained from Changsha Tianqin Biotechnology Co., Ltd (Changsha, China). Animals were raised under a 12/12 h day/night cycle at a temperature of 20-22 °C. They received a standard pellet diet and water *ad libitum*. All experimental projects were approved by the Animal Experimental Ethics Committee of Wannan Medical College.

### Experimental design

**Figure 1:**
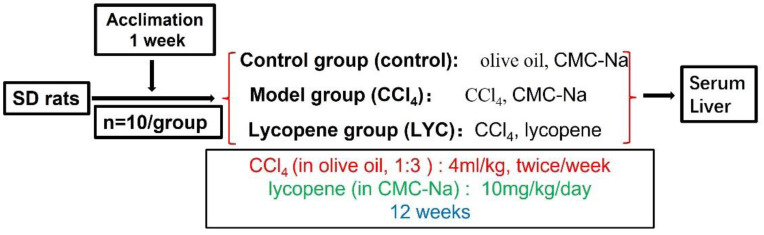
Experimental protocol. CMC-Na - sodium carboxymethyl cellulose; CCl_4_ - carbon tetrachloride; SD - Sprague-Dawley.

Lycopene was administered as previously described (12,30). The experiment was performed as shown in Figure 1. After 1-week acclimatization, the rats were weighed and numbered by body weight from light to heavy and then divided into three groups (10 per group) according to methods for randomly grouping experimental animals. In the control group, the rats were treated with olive oil twice per week for 12 weeks. In the CCl_4_ group, the rats were treated with 4 mL/kg of CCl_4_ (dissolved in olive oil) via intraperitoneal injection twice per week for 12 weeks. In the lycopene group, the animals were treated with 4 mL/kg of CCl_4_ (1/3 = v/v, dissolved in olive oil) and orally administered lycopene (10 mg/kg-BW/d) twice per week for 12 weeks. At the end of the experiment, fasting blood samples were collected, and liver tissues were removed. A part of the liver from each rat was stored at -80 °C, and the rest was fixed in 10% neutral formalin for histological analysis. The epididymal adipose tissue was separated and weighed to assess the amount of visceral fat.

### Determination of liver function markers

Fasting blood samples were centrifugated at 3000 g at 4 °C for 15 min to separate the serum. AST and ALT levels in serum were determined for the assessment of liver function with an automated bio-chemical analyzer.

### Determination of inflammatory cytokines

Liver tissue was homogenized in phosphate buffered saline (PBS) and centrifuged for 15 min at 12 000 g for separation of the liver homogenates. TNF-α and IL-6 levels in the liver homogenates were measured with specific ELISA kits (Hefei Bomei Biotechnology co., LTD, Hefei, China) in accordance with the manufacturer’s protocol.

### Measurement of oxidative stress biomarkers

The activities of catalase (CAT) and superoxide dismutase (SOD) in the livers were assessed with reagent kits (Nanjing Jiancheng Bioengineering Institute, Nanjing, China). The thiobarbituric acid method was used to measure malondialdehyde (MDA) level with a diagnostic kit (Nanjing Jiancheng Bioengineering Institute) according to the manufacturer’s protocol. MDA level was determined by detecting absorbance at 532 and 450 nm.

### Histological examination

After dehydration, fixed liver tissues were embedded in paraffin, cut into 5 μm-thick sections, and stained with hematoxylin-eosin (H-E). The histological features of liver tissues and cell injury were observed under a light microscope (Olympus, Tokyo, Japan). The sections stained with Masson’s trichrome were used to evaluate collagen expression by calculating the percentage of the stained area in the total area.

### Immunohistochemical analysis

After deparaffinization with xylene and hydration, the sections were immersed into 10-mM sodium citrate buffer for antigen retrieval. Subsequently, the sections were incubated with hydrogen peroxide for 15 min to inhibit endogenous peroxidase activity. After incubation with bovine serum albumin (dissolved in PBS) to block nonspecific sites, the sections were immersed in PBS containing anti-α-SMA (1:100) and anti- TGF-β1 (1:100) antibody overnight at 4 °C. The sections were rinsed with PBS and treated with secondary antibody for 60 min. The antigen was detected by visualization with 3, 3′-diaminobenzidine (DAB) streptavidin-horseradish peroxidase substrate kit.

### Western blotting

Liver tissues were lysed with ice-cold lysis buffer and centrifuged at 12 000 g for 20 min at 4 °C for supernatant separation. The supernatant was used to determine the amount of protein with a BCA kit (Yanjing Biotechnology, Shanghai, China). Equal amounts of protein were separated with sodium dodecyl sulfate sample buffer. The protein in the samples was electrophoretically isolated on 10% sodium dodecyl sulfate polyacrylamide gel and transferred to nitrocellulose membranes. The membranes were blocked with 5% non-fat milk dissolved in TBS-T (50 mM Tris 150 mM NaCl, 0.05% Tween-20) for 1 h and then hybridized with anti-β-actin, HO-1, SIRT1, IκB α, p-IκB α, NF-κB, p-NF-κB, LC3, P62, Beclin 1, REDD1, SHP2, PGC 1α, TGF-β1, and α-SMA antibodies (dissolved in TBS-T buffer containing 5% non-fat milk) overnight at 4 °C. The membranes were washed with TBS-T and incubated with a peroxidase-conjugated secondary antibody for 90 min. The protein bands were visualized on the membrane by DAB kit and analyzed with ImageJ software (version 1.8; National Institutes of Health, Bethesda, MD, USA).

### Statistical analysis

The data are expressed as means ± standard deviation (SD). The normality of distribution was tested with a Shapiro-Wilk test. The significance of differences between two groups was assessed with one-way analysis of variance (ANOVA) followed by a Tukey *post-hoc* test. A *P* value of lower than 0.05 was considered statistically significant. The analysis was conducted with SPSS, version 22.0 (IBM Corp., Armonk, NY, USA).

## RESULTS

### Changes of body weight, liver weight, and liver weight/body weight ratio

**Figure 2:**
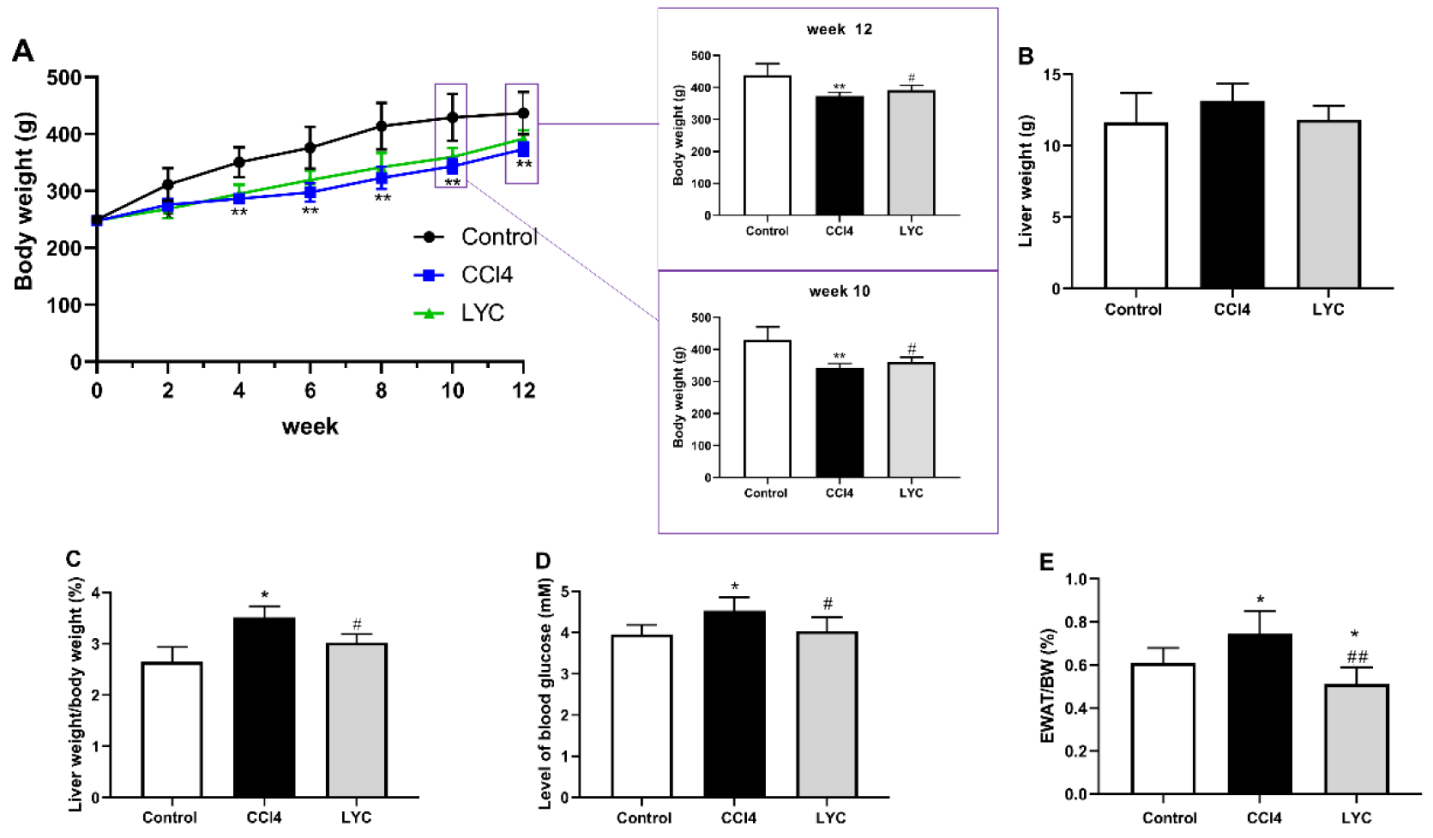
The effect of lycopene on carbon tetrachloride (CCl_4_) -induced symptoms in rats. (**A**) Body weight. (**B**) Liver weight. (**C**) Ratio of liver weight to body weight. (**D**) Fasting blood glucose. (**E**) Epididymal white adipose tissue (EWAT). LYC - lycopene group. *P < 0.05 and **P < 0.01 compared with the control group. ^$^*P* < 0.05 and ^$$^*P* < 0.01 compared with the CCl_4_ group. Data are presented as the mean values ± standard deviation. Eight rats in each group.

First, we examined the effect of lycopene on the features of the rats treated with CCl_4_. After two weeks of exposure to CCl_4_, body weight of the rats in the CCl_4_ and lycopene groups was significantly lower than in the control group (*P* < 0.05, Figure 2). But body weight in the lycopene group did not significantly differ from that in the CCl_4_ group before the tenth week (*P* > 0.05), and it significantly increased in the 10th (359.9 ± 15.91 vs 343.5 ± 12.48 g, *P* = 0.034) and 12th week (391.7 ± 14.72 vs 373.5 ± 11.40 g, *P* = 0.013) (Figure 2A). Furthermore, there were no significant differences in liver weight between the groups (*P* > 0.05) (Figure 2B). However, liver weight-to-body weight ratio was significantly higher in the CCl_4_ group than in the control group (Figure 2C) (3.53 ± 0.22% vs 2.65 ± 0.29%, *P* < 0.001), and lycopene treatment decreased the ratio when compared with the CCl_4_ group (Figure 2C) (3.06 ± 0.20% vs 3.53 ± 0.22%, *P* < 0.001). The level of fasting blood glucose in the CCl_4_ group was higher than that in the control and lycopene group (4.53 ± 0.33 vs 3.96 ± 0.23 mmol/L, *P* = 0.0014), but the levels of fasting blood glucose in every group were normal (Figure 2D). CCl_4_ treatment increased fat accumulation in the epididymis when compared with the control group (0.746 ± 0.106% vs 0.610 ± 0.069%, *P* = 0.011) (Figure 2E). However, lycopene administration inhibited fat accumulation in the epididymis compared with the CCl_4_ group (0.511 ± 0.077% vs 0.746 ± 0.106%, *P* < 0.001) (Figure 2E).

### The effect of lycopene on liver function

**Figure 3:**
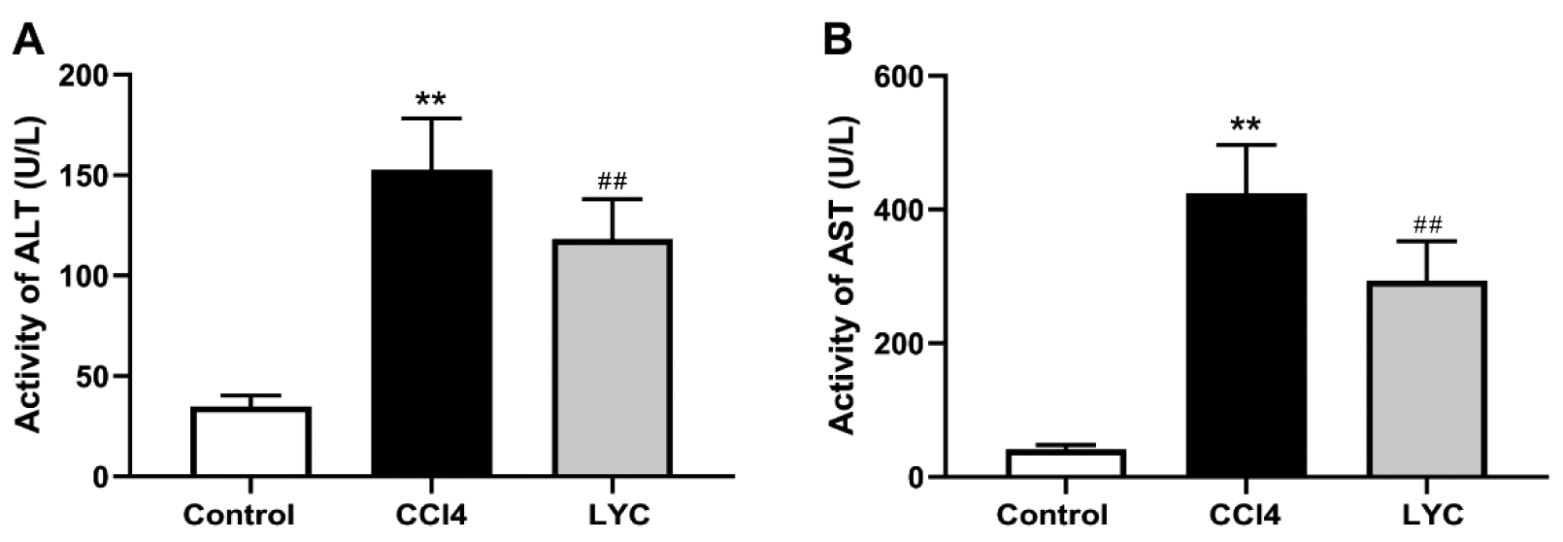
The effect of lycopene on liver function in rats that underwent carbon tetrachloride (CCl_4_) treatment (**A**) Alanine transaminase (ALT). (**B**) Aspartate transaminase (AST). LYC - lycopene group. *P < 0.05 and **P < 0.01 compared with the control group. ^#^*P* < 0.05 and ^##^*P* < 0.01 compared with the CCl_4_ group. Data are presented as the mean values ± standard deviation. Eight rats in each group.

CCl_4_ administration elevated the activities of ALT (152.5 ± 25.70 vs 34.9 ± 5.44 U/L, *P* < 0.001) and AST (424.88 ± 72.04 vs 41.4 ± 6.57 U/L, *P* < 0.001) compared with the control group (Figure 3). Lycopene administration decreased the activities of ALT (118.3 ± 19.77 vs 152.5 ± 25.70 U/L, *P* = 0.010) and AST (293.5 ± 59.16 vs 424.88 ± 72.04 U/L, *P* = 0.0014) compared with the CCl_4_ group (Figure 3).

### The effect of lycopene on CCl_4_-induced liver fibrosis

**Figure 4:**
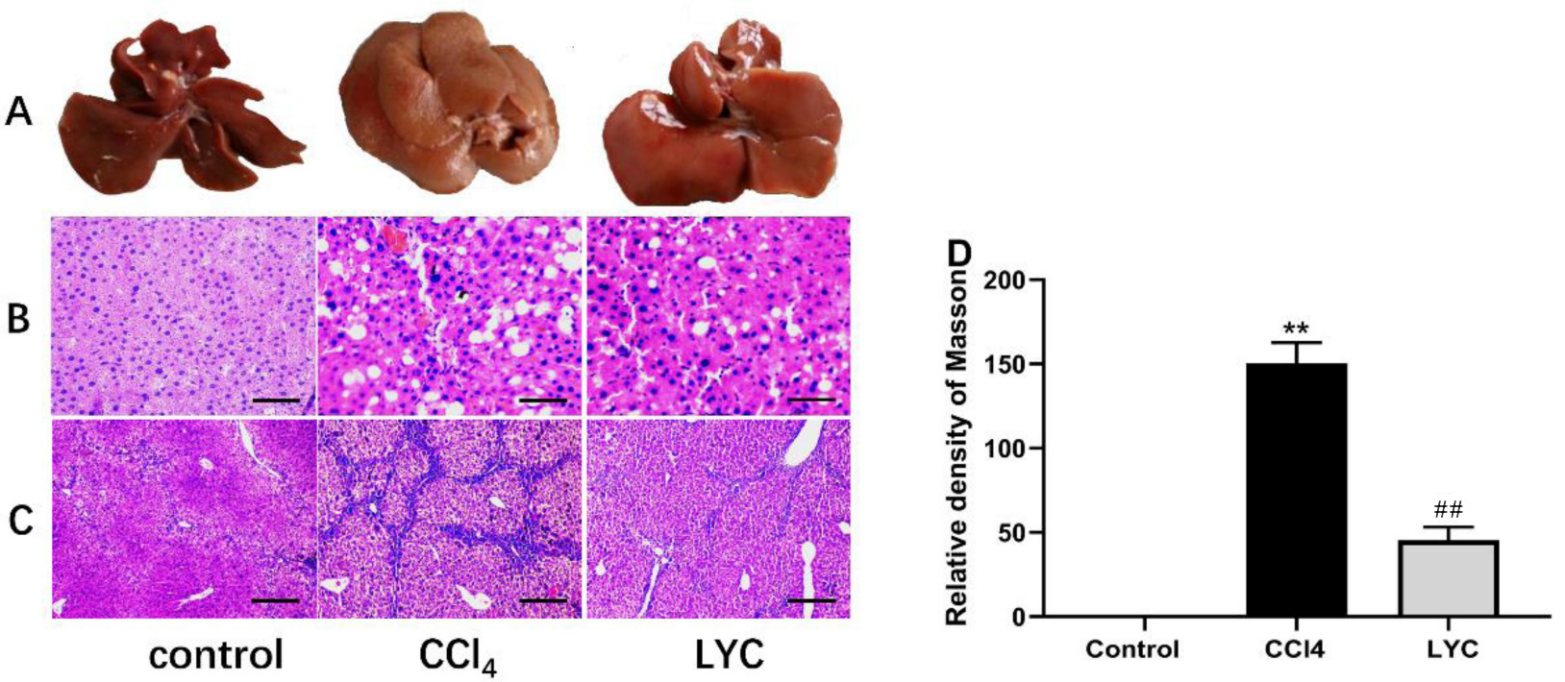
The effect of lycopene on liver fibrosis in rats. (**A**) The livers at the end of the experiment. (**B**) The livers stained with hematoxylin and eosin. (**C**) The livers stained with Masson’s trichrome. (**D**) Score of Masson’s trichrome staining. LYC - lycopene group. **P < 0.01 compared with the control group. ^$$^*P* < 0.01 compared with the carbon tetrachloride (CCl_4_) group. Data are presented as the mean values ± standard deviation. Eight rats in each group.

Macroscopic observation suggested that the livers exposed to CCl_4_ presented typical characteristics of fibrosis, and that lycopene treatment significantly ameliorated fibrosis induced by CCl_4_ (Figure 4A). Histological observation showed that hepatocytes in the CCl_4_ group were larger than in the control group. In the liver tissue of the CCl_4_ group, diffuse small nodules were observed (Figure 4B). CCl_4_ administration led to inflammatory infiltration and impairment of lobular architecture, and hepatic cords were irregular (Figure 4B). Lycopene treatment obviously decreased the necrosis of hepatic cells and collagen deposition in the liver tissue when compared with the CCl_4_ group (Figure 4B).

The extent of hepatic fibrosis is closely related to the generation of collagen fibers. CCl_4_ treatment significantly increased the density of Masson staining compared with the control group, while lycopene treatment decreased it compared with the CCl_4_ group (Figure 4C, 4D). These results suggested that CCl_4_ treatment markedly increased the deposition of collagen fibers in the liver tissue compared with the control group (Figure 4C) and that lycopene treatment largely reduced this deposition (Figure 4C).

### The effect of lycopene on the expression of profibrogenic cytokines TGF-β1 and α-SMA

**Figure 5:**
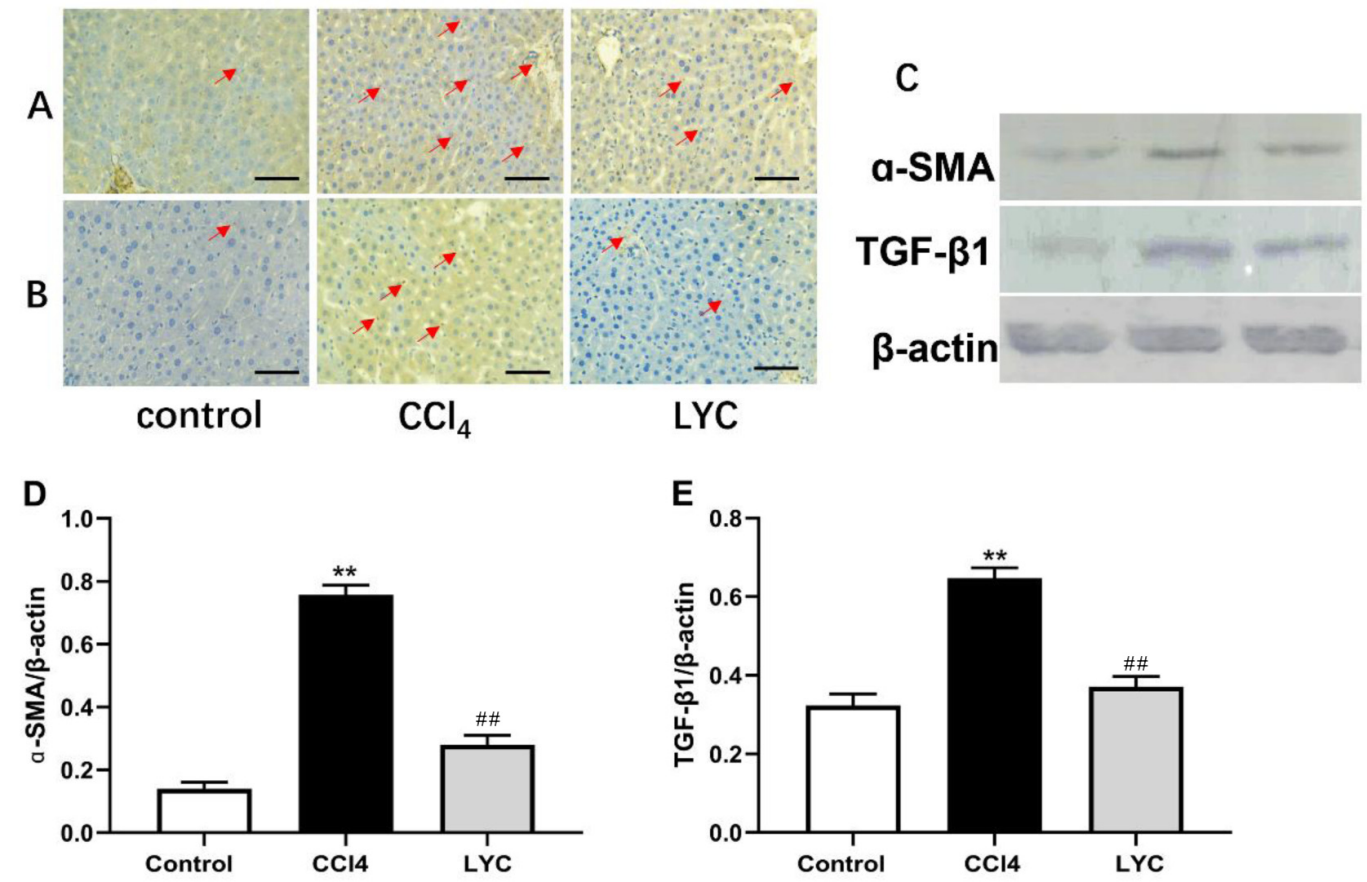
The effect of lycopene on the expression of α-smooth muscle actin (α-SMA) and transforming growth factor β1 (TGF-β1) protein. (**A**) Immunohistochemical staining of α-SMA. (**B**) Immunohistochemical staining of TGF-β1. (**C**) Representative Western blots. (**D**) Quantitative analysis for α-SMA. (**E**) Quantitative analysis for TGF-β1. LYC - lycopene group. **P < 0.01 compared with the control group. ^##^*P* < 0.01 compared with the carbon tetrachloride (CCl_4_) group. Data are presented as the mean values ± standard deviation. Eight rats in each group.

HSCs are activated by TGF-β1, a key profibrogenic cytokine, and generate α-SMA, which is regarded as a marker of activated HSCs (7). Immunohistochemical staining showed that CCl_4_ treatment significantly increased the expression of TGF-β1 and α-SMA compared with the control group. Lycopene decreased α-SMA and TGF-β1 staining (Figure 5A, 5B). Western blotting showed that CCl_4_ significantly elevated the expression of α-SMA and TGF-β1 protein when compared with the control group (Figure 5C-E). However, lycopene reduced this expression when compared with the CCl_4_ group (Figure 5C-E).

### The effect of lycopene on oxidative stress and inflammation response

**Figure 6:**
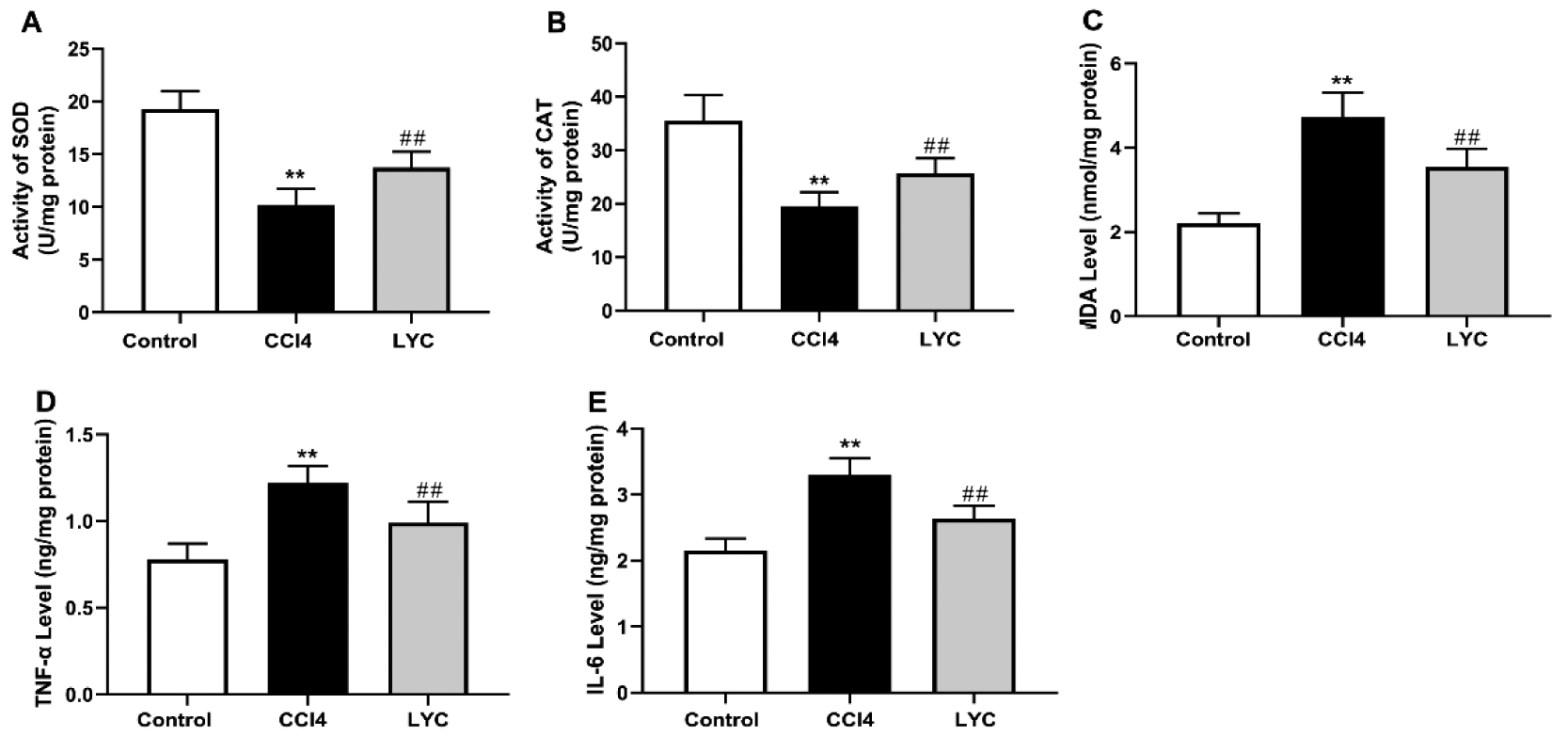
Changes of antioxidation and inflammatory cytokines in the livers. (**A**) Activity of superoxide dismutase (SOD). (**B**) Activity of catalase (CAT). (**C**) Level of malondialdehyde (MDA). (**D**) Level of tumor necrosis factor-α (TNF-α). (**E**) Level of interleukin 6 (IL-6). LYC - lycopene group. **P < 0.01 compared with the control group. ^##^*P* < 0.01 compared with the carbon tetrachloride (CCl_4_) group. Data are presented as the mean values ± standard deviation. Eight rats in each group.

To investigate the mechanisms of action of lycopene in CCl_4_-induced hepatic fibrosis, we assessed oxidative stress and inflammation. Compared with the control group, CCl_4_ elevated the levels of TNF-α (1.22 ± 0.098 vs 0.78 ± 0.091 ng/mg protein, *P* < 0.001), IL-6 (3.30 ± 0.26 vs 2.15 ± 0.18 ng/mg protein, *P* < 0.001), and MDA (4.73 ± 0.58 vs 2.21 ± 0.23 nmol/mg protein, *P* < 0.001), and reduced SOD (10.20 ± 1.56 vs 19.30 ± 1.69 U/mg protein, *P* < 0.001) and CAT activities (19.53 ± 2.66 vs 35.54 ± 4.82 U/mg protein, *P* < 0.001) (Figure 6). Compared with the CCl_4_ group, lycopene administration decreased the levels of TNF-α (0.99 ± 0.122 vs 1.22 ± 0.098 ng/mg protein, *P* = 0.001), IL-6 (2.63 ± 0.20 vs 3.30 ± 0.26 ng/mg protein, *P* < 0.001), and MDA (3.55 ± 0.44 vs 4.73 ± 0.58 nmol/mg protein, *P* < 0.001), and increased SOD (13.75 ± 1.48 vs 10.20 ± 1.56 U/mg protein, *P* < 0.001) and CAT (25.73 ± 2.77 vs 19.53 ± 2.66 U/mg protein, *P* < 0.001) activities.

**Figure 7:**
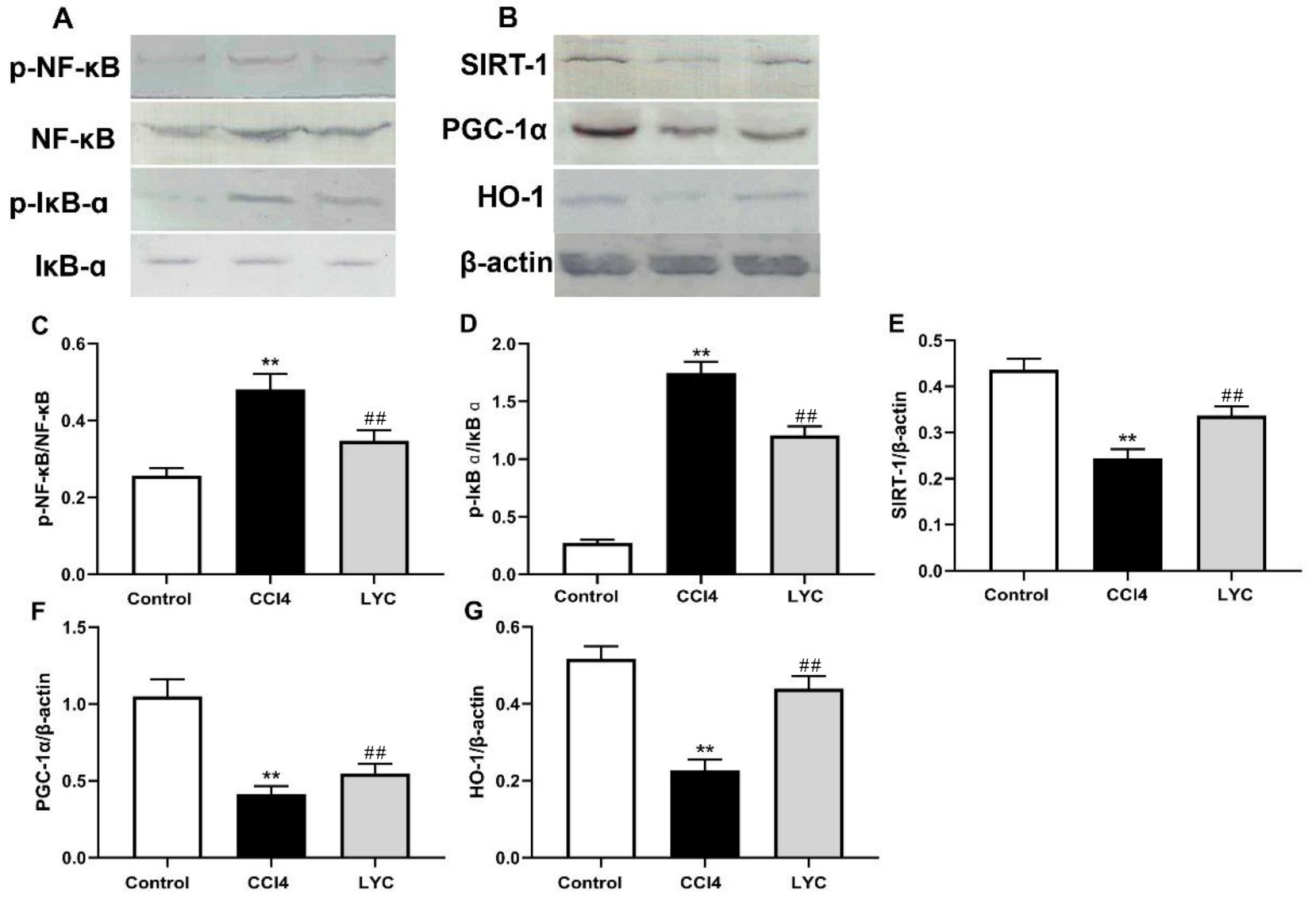
The effect of lycopene on nuclear factor κB (NF-κB) and inhibitor of NF-κB α (IκB-α) phosphorylation (**A**), and the expression of sirtuin 1 (SIRT1), peroxisome proliferator-activated receptor gamma coactivator 1α (PGC-1α), and heme oxygenase 1 (HO-1) (**B**) in the liver. (**C**) Quantitative analysis of p-NF-κB; (**D**) p-IκB-α; (**E**) sirtuin 1 (SIRT-1); (**F**) PGC-1α; and (**G**) heme oxygenase 1 (HO-1). LYC - lycopene group. **P < 0.01 compared with the control group. ^##^*P* < 0.01 compared with the carbon tetrachloride (CCl_4_) group. Data are presented as the mean values ± standard deviation. Eight rats in each group.

To investigate the change of inflammatory response, the levels of phosphorylated NF-κB and IκB-α were measured with Western blotting (Figure 7A). CCl_4_ treatment increased NF-κB and IκB-α phosphorylation in the liver tissue compared with the control group (*P* < 0.05) (Figure 7C, 7D), and lycopene decreased it compared with the CCl_4_ group (*P* < 0.05) (Figure 7C, 7D).

The activation of the SIRT 1/PGC 1α/HO-1 axis was demonstrated to reduce oxidative stress and inflammation (31,32). Thus, Western blotting was used to determine the expression of HO-1, PGC 1α, and SIRT 1 in the liver (Figure 7B). The expression of HO-1, PGC 1α, and SIRT 1 in the liver was inhibited by CCl_4_ treatment compared with the control group (*P* < 0.05) (Figure 7E-G). However, lycopene administration significantly increased the expressions of HO-1, PGC 1α, and SIRT 1 protein compared with the CCl_4_ group (*P* < 0.05) (Figure 7E-G).

### The effect of lycopene on autophagy-related protein expression

**Figure 8:**
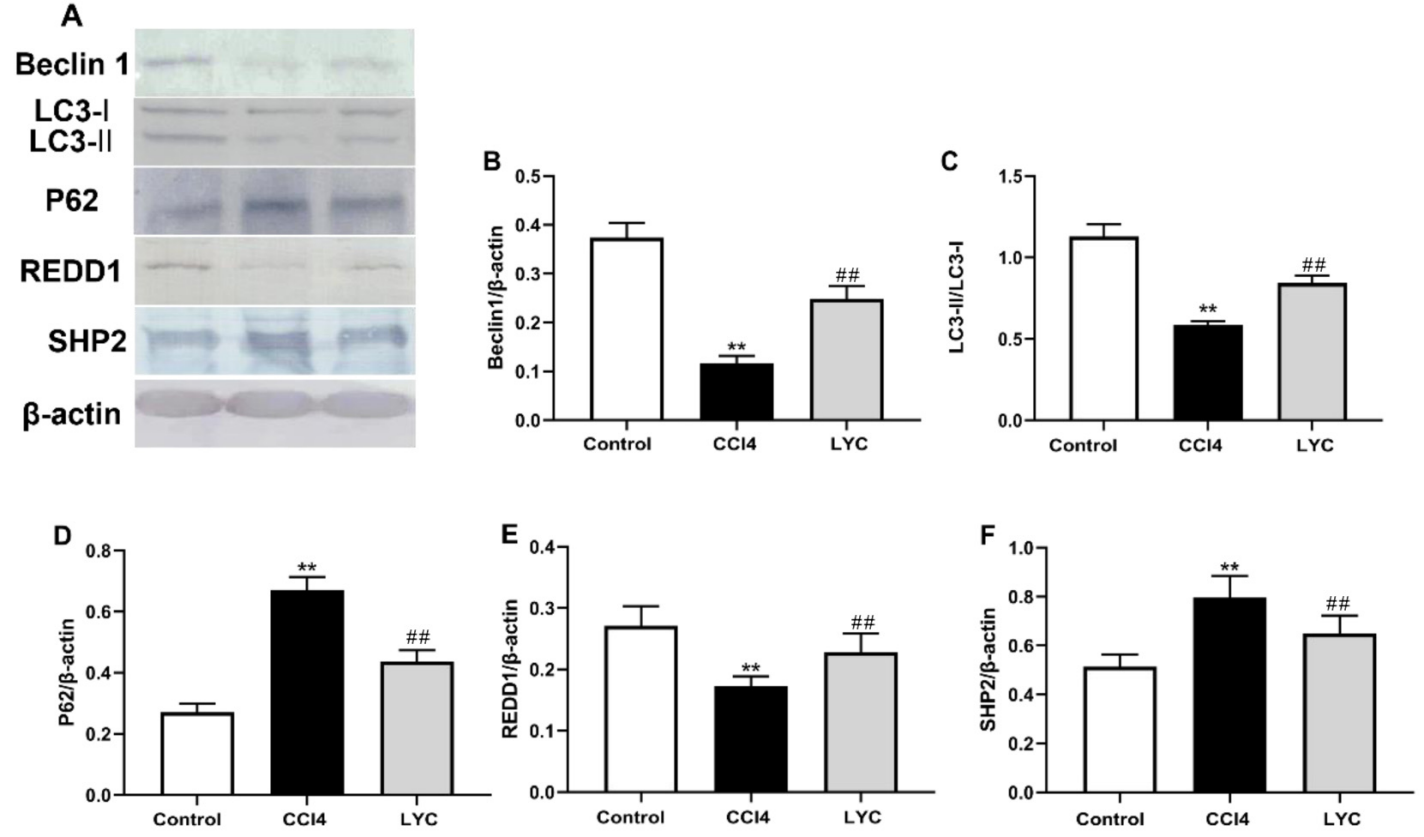
The effect of lycopene on autophagy pathway. (**A**) Representative Western blots. (**B**) Quantitative analysis of beclin 1 (**C**); the ratio of microtubule-associated proteins 1 light chain 3 (LC3)-II to LC3-I; (**D**) P62; (**E**) regulated in development and DNA damage responses 1 (REDD1); and (**F**) src homology 2-containing protein tyrosine phosphatase (SHP)-2. LYC - lycopene group. **P < 0.01 compared with the control group. ^##^*P* < 0.01 compared with the carbon tetrachloride (CCl_4_) group. Data are presented as the mean values ± standard deviation. Eight rats in each group.

To further elucidate the potential mechanisms of action of lycopene on liver fibrosis, the expression of LC3, Beclin 1, and P62 in the liver was determined with Western blotting (Figure 8A). CCl_4_ reduced Beclin 1 expression and LC3 II/I ratio, and increased P62 expression compared with the control group (Figure 8B-D). Lycopene countered the effect of CCl_4_ on the ratio of LC3 II/I and Beclin 1 and P62 expression (Figure 8B-D). Additionally, lycopene increased the expression of REDD1 (Figure 8E) and reduced SHP2 level (Figure 8F) in the liver.

## DISCUSSION

**Figure 9:**
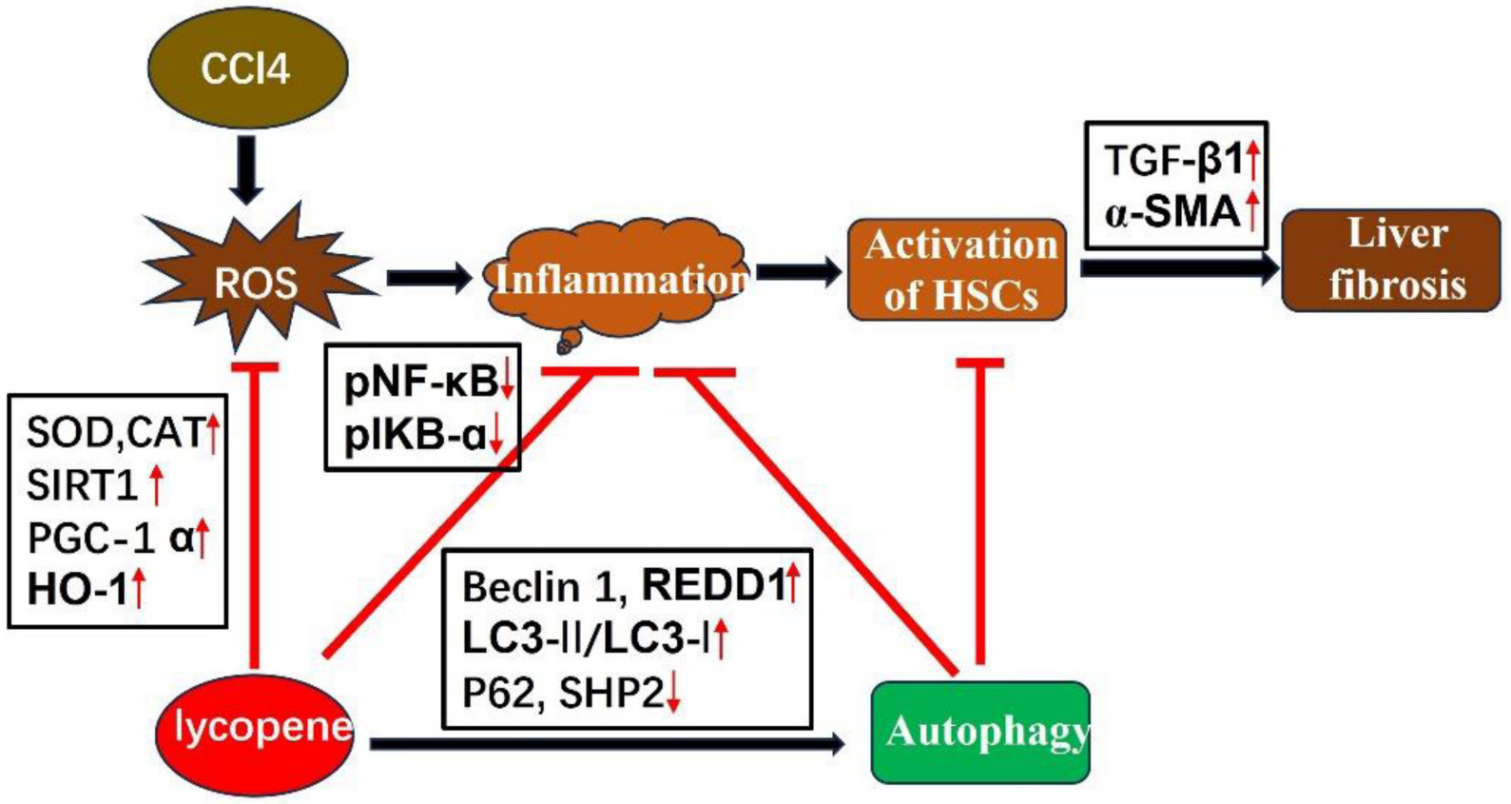
Probable mechanisms underlying the effect of lycopene on liver fibrosis. CCl_4_ - carbon tetrachloride; ROS - reactive oxygen species; HSC - hepatic stellate cells; α-SMA - α-smooth muscle actin; TGF-β1- transforming growth factor β1; SOD - superoxide dismutase; CAT - catalase; SIRT1 - sirtuin 1; PGC-1α - peroxisome proliferator-activated receptor gamma coactivator 1α; HO-1 - heme oxygenase 1; REDD1 - regulated in development and DNA damage responses 1; LC3 - microtubule-associated protein 1 light chain 3B; SHP-2 - src homology 2-containing protein tyrosine phosphatase-2.

In this study, lycopene attenuated CCl_4_-induced hepatic fibrosis. Probable mechanisms underlying this effect are shown in Figure 9.

Lycopene exhibits a hepatoprotective effect against bisphenol A-induced toxicity and inhibits HSC activation *in vitro* (12,19). In the current study, lycopene decreased ALT and AST levels, thereby attenuating CCl_4_-induced liver injury. It also reduced the collagen fiber deposition in the liver of rats injected with CCl_4_. However, the results suggested that lycopene attenuated hepatic fibrosis via reducing HSC activation.

Chronic oxidative stress is a key factor in initiating hepatic fibrosis (33). Exposure to CCl_4_ can damage various tissues, primarily the liver (34,35), as CCl_4_ induces oxidative stress and inflammatory response. CCl_4_ can be converted into free radicals by CYP2E1 in the liver following ROS generation, a mechanism impairing the liver cell membrane (36). Liver injury resulting from lipid peroxidation and excess production of ROS contributes to inflammatory response and causes a release of pro-inflammatory factors such as TNF-α, IL-1β, and IL-6, thus exacerbating liver injury (37). Excessive ROS and the ensuing inflammatory responses initiate liver fibrosis by triggering the generation of pro-fibrogenic mediators such as TGF-β1 and HSCs activation (38,39). The effects of many antioxidants, including natural products, on liver fibrosis have been well elucidated, and some of these antioxidants have been tested in clinical trials (40,41).

Lycopene can ameliorate the progression of a wide range of disorders such as neurodegenerative disorders (42) and heart failure (43) via its strong anti-oxidative and anti-inflammatory effects attributed to its highly unsaturated double bonds. In the present study, oral administration of lycopene increased SOD activity and reduced the levels of MDA, IL-6, and TNF-α. Furthermore, lycopene treatment reduced the levels of phosphorylated NF-κB and IκB-α. NF-κB plays an important role in modulating the development of inflammation. A release of inflammatory factors such as TNF-α and IL-6 is associated with the activation of the NF-κB/IκB α pathway. The NF-κB/IκB α pathway is also considered a redox-sensitive pathway involved in the development of liver fibrosis (44). Our results indicated that lycopene alleviated liver fibrosis through antioxidant and anti-inflammatory effects.

SIRT1 exerts its eminent cytoprotective effect via deacetylating peroxisome PGC-1α and forkhead box transcription factor, and via stimulating the expression of antioxidative enzymes, including HO-1 (31). HO-1, an enzyme induced by multiple stress factors, catabolizes the conversion of heme into bilirubin, carbon monoxide, and iron (45). HO-1 and its catalystic products ameliorate tissue injury via antioxidation, anti-inflammation, and anti-apoptotic effect in various diseases (46-48). Activated SIRT1 inhibits liver fibrosis and activation of HSCs induced by CCl_4_ (27). Our study showed that CCl_4_ exposure reduced the expression of HO-1, SIRT1, and PGC-1α, while lycopene treatment increased their expression. These results suggested that SIRT1/PGC-1α/HO-1 signaling is involved in the antioxidant effect of lycopene.

When autophagy is triggered, LC3 is successively changed into LC3-I and LC3-II. Furthermore, LC3-I is enzymatically processed into LC3-II, which is involved in the formation of autophagosome. Therefore, the ratio of LC3-II to LC3-I indicates the level of autophagy (49). In addition, Beclin-1 and P62 (SQSTM1) play a key role in the regulation of autophagy, and P62 negatively regulates autophagy activity (50). Beclin-1, one of the markers of autophagy, modulates autophagosomal membrane nucleation via regulating the expression of autophagy proteins (50). Interestingly, the effect of autophagy in liver fibrosis is controversial. On the one hand, activation of autophagy promotes liver fibrosis via activating HSCs (51,52); however, upregulated autophagy could improve liver fibrosis through an anti-inflammatory effect (23,53). Another study found that autophagy increased the activation of quiescent HSCs. Activation of autophagy was also found to cause the senescence of activated HSCs and collagen degradation, and inhibit inflammation, attenuating liver fibrosis (54). Many natural products have been confirmed to alleviate liver fibrosis by ameliorating autophagy (53,55). SHP2 upregulated mammalian target of rapamycin (mTOR), stimulates liver injury and fibrosis, and reduces autophagy (56). Furthermore, REDD1 is involved in autophagy via mTOR signaling, and the knockdown of SHP2 increases REDD1 expression, reduces the expression of α-SMA induced by CCl_4_ in mice, and attenuates liver fibrosis (57). In the present study, lycopene treatment increased the LC3-II/I ratio and Beclin-1 and REDD1 expression, and reduced P62 and SHP2 expression. These results indicated an ameliorating effect of lycopene on autophagy. The exposure to CCl_4_ has been confirmed to trigger TGF-β1generation and HSCs activation (38,39). However, the amelioration of autophagy by lycopene promoted the senescence of activated HSCs, which further retarded the development of liver fibrosis.

Our study has several limitations. First, the mechanisms underlying the effect of lycopene against liver fibrosis need to be further clarified. For example, the effect of inhibition of SIRT 1/PGC-1α/HO-1 signaling on liver fibrosis should be investigated. Second, the mechanisms underlying the effect of lycopene on liver fibrosis should be explored in more detail through an *in vitro* study. In addition, studies with larger sample sizes should be used to elucidate the mechanisms on a long-term basis.

In summary, the present findings showed that lycopene treatment delayed the progression of CCl_4_-induced liver fibrosis in rats. The beneficial effect of lycopene on liver fibrosis may be associated with its inhibition of oxidative stress and inflammation, and amelioration of autophagy in rats.
